# Baroreflex Modulation During Acute High-Altitude Exposure in Rats

**DOI:** 10.3389/fphys.2020.01049

**Published:** 2020-08-21

**Authors:** Ana Rosa Beltrán, Alexis Arce-Álvarez, Rodrigo Ramirez-Campillo, Manuel Vásquez-Muñoz, Magdalena von Igel, Marco A. Ramírez, Rodrigo Del Rio, David C. Andrade

**Affiliations:** ^1^Departamento de Educación, Facultad de Educación, Universidad de Antofagasta, Antofagasta, Chile; ^2^Laboratorio de Fisiología Celular, Departamento Biomédico, Facultad de Ciencias de la Salud, Universidad de Antofagasta, Antofagasta, Chile; ^3^Escuela de Kinesiología, Facultad de Salud, Universidad Católica Silva Henríquez, Santiago, Chile; ^4^Laboratory of Human Performance, Quality of Life and Wellness Research Group, Department of Physical Activity Sciences, Universidad de Los Lagos, Osorno, Chile; ^5^Centro de Investigación en Fisiología del Ejercicio, Facultad de Ciencias, Universidad Mayor, Santiago, Chile; ^6^Laboratory of Cardiorespiratory Control, Department of Physiology, Pontificia Universidad Católica de Chile, Santiago, Chile; ^7^Centro de Envejecimiento y Regeneración (CARE), Pontificia Universidad Católica de Chile, Santiago, Chile; ^8^Centro de Excelencia en Biomedicina de Magallanes (CEBIMA), Universidad de Magallanes, Punta Arenas, Chile; ^9^Pedagogía en Educación Física, Deportes y Recreación, Universidad Mayor, Santiago, Chile

**Keywords:** baroreflex, autonomic nervous system, sympathetic nervous system, parasympathetic nervous system, baroreceptors

## Abstract

Baroreflex (BR) control is critically dependent of sympathetic and parasympathetic modulation. It has been documented that during acute hypobaric hypoxia there is a BR control impairment, however, the effect of a natural hypoxic environment on BR function is limited and controversial. Therefore, the aim of this study was to determine the effect of acute High-Altitude exposure on sympathetic/parasympathetic modulation of BR control in normal rats. Male Sprague Dawley rats were randomly allocated into Sea-Level (*n* = 7) and High-Altitude (*n* = 5) (3,270 m above sea level) groups. The BR control was studied using phenylephrine (Phe) and sodium nitroprusside (SNP) through sigmoidal analysis. The autonomic control of the heart was estimated using heart rate variability (HRV) analysis in frequency domain. Additionally, to determine the maximum sympathetic and parasympathetic activation of BR, spectral non-stationary method analysis, during Phe (0.05 μg/mL) and SNP administration (0.10 μg/mL) were used. Compared to Sea-Level condition, the High-Altitude group displayed parasympathetic withdrawal (high frequency, 0.6–2.4 Hz) and sympathoexcitation (low frequency, 0.04–0.6 Hz). Regarding to BR modulation, rats showed a significant decrease (*p* < 0.05) of curvature and parasympathetic bradycardic responses to Phe, without significant differences in sympathetic tachycardic responses to SNP after High-Altitude exposure. In addition, the non-stationary analysis of HRV showed a reduction of parasympathetic activation (Phe) in the High-Altitude group. Our results suggest that acute exposure to High-Altitude produces an autonomic and BR control impairment, characterized by parasympathetic withdrawal after 24 h of high-altitude exposure.

## Introduction

The cardiac baroreflex (BR) function governs short-term fluctuations of blood pressure and, therefore, plays an important role in the regulation of several homeostatic functions (Stauss, 2002). The main baroreceptors are allocated in the aortic arc and the carotid sinus area (Stauss, 2002). The increase of blood pressure (BP) triggers a BR-dependent increase of parasympathetic drive, while a decrease of BP produces a massive sympathoexcitation (Cowley and Guyton, 1975). It has been demonstrated that modification of this reflex arc is implicated in several physiological and pathophysiological conditions, such as microgravity, aging processes, hypertension, heart failure, High-Altitude exposure, among others (Ferguson et al., 1992; Wang et al., 2004; Hainsworth et al., 2007; Monahan, 2007; Eckberg et al., 2010; Del Rio et al., 2013, 2016; Fernandez et al., 2015; Andrade et al., 2017, 2019).

Among the aforementioned conditions, High-Altitude is amongst the most inhospitable environments on earth and it has been demonstrated that exposure to hypobaric hypoxia is strongly related to impairment of autonomic control (Lanfranchi et al., 2005; Hainsworth et al., 2007). Although it has been observed that BR control is compromised during hypobaric hypoxia, the evidence is limited and controversial; nevertheless, the major differences related to autonomic modulation could be related to the atmospheric pressure (Sagawa et al., 1997; Hainsworth et al., 2007). Human studies, using the neck chamber method, showed that hypobaric hypoxia had no effect on the BR set point, but reduce the BR gain (Sagawa et al., 1997). Contrarily, it has been shown that during normobaric hypoxia, the BR gain was not modified (Bourdillon et al., 2017). In addition, Obrezchikova et al. (2000) showed that the exposure to chronic hypobaric hypoxia (hypoxic chamber) inhibits vagal bradycardia BR in rats. Despite the fact, that these evidences strongly suggest that BR control is affected during hypoxic environment, it does not necessarily have to be reproducible during natural conditions (i.e., High-Altitude environment). Indeed, most studies address the contribution of sympathetic modulation of the BR function during hypobaric hypoxia simulating High-Altitude ambient (Hainsworth et al., 2007; Simpson et al., 2019), but none of these focused on the estimation of the parasympathetic contribution on BR modulation. Considering that there are few evidences underpinning the acute effect of high-altitude exposure on BR control, we proposed to determine the effect of acute high-altitude environment (3,270 m above sea level) on sympathetic/parasympathetic modulation of BR control in normal rats.

## Materials and Methods

### Ethical Approval and Animals

Twelve male Sprague-Dawley rats were used in these experiments. All surgical procedures and protocols used, were in accordance with guidelines of the American Physiological Society and the National Institutes of Health Guide for the Care and Use of Laboratory Animals and were approved by the University of Antofagasta Scientific Research Ethical Committee (CEIC-210/2019).

### Experimental Procedure

Male Sprague-Dawley rats (*n* = 12) were housed in individual cages with a 12/12-h light/dark schedule and were allowed free access to food and water. The rats were randomly allocated into Sea-Level group (*n* = 7) and to High-Altitude group (*n* = 5). Sea-level rats were subjected to catheterization surgery according to the method of Li et al. (1999) and basal BP recording was preformed (1-hour). Afterward, BR experiment was performed as follows: 8 boluses of phenylephrine to increase BP were injected (*i.v.*) and after 30 min of recovery, 8 boluses of sodium nitroprusside to decrease BP were injected (*i.v.*). The second series of rats (High-Altitude group) ascended at 3,270 m above sea level (Caspana, Antofagasta, Chile) in a costume made mobile laboratory and after 24 h, the catheterization surgery was performed. Similar to the first animal series (Sea-Level group), 8 h after (Masson et al., 2014) the surgical procedure, basal recordings of BP (1-hour) and the BR experiment were performed. At Sea-Level the relative humidity was between 66 and 68% and the temperature between 19 and 21°C, while at 3,270 m (High-Altitude), the relative humidity was between 21 and 25% and the temperature was 19°C (Chilean Meteorological Service).

### Arterial Blood Pressure in Freely Moving Rats

Arterial BP measurement was performed in conscious freely moving rats. The carotid artery and jugular vein cannulations (PE-50 polyethylene tubing, Clay Adams, Parsippany, NJ, United States), were performed to measure BP and for drugs administration. The rats were anesthetized (*i.p.*) using ketamine (80 mg/kg; Fort Dodge Animal Health, United States) plus xylazine (12 mg/kg; Alcon, United States) (Li et al., 1999; Feng et al., 2015). A midline incision in the neck was performed to isolate a lateral branch of the carotid artery. A small incision was made and a 3 Fr polyurethane catheter was guided into the artery and was tunneled subcutaneously to the back of the neck and connected to a vascular access port. Eight hours before BP measurement, the rats were anesthetized (i.p.) using ketamine (80 mg/kg; Fort Dodge Animal Health, United States) plus xylazine (12 mg/kg; Alcon, United States) for catheterization of the common carotid artery and jugular vein (Li et al., 1999; Feng et al., 2015). The BP was continuously recorded in a BIOPAC system (DA100C, BIOPAC system, United States) at a sampling rate of 1 KHz. From recordings we were able to estimate systolic blood pressure (SBP), diastolic blood pressure (DBP), pulse pressure (PP = SBP–DBP) and mean arterial blood pressure (MABP = 1/3 of SBP + 2/3 of DBP). In addition, the heart rate (HR) was derived from dP/dt signal obtained from the BP recordings (Del Rio et al., 2016; Andrade et al., 2017).

### Baroreflex Control

The BR was evaluated by repeated bolus injections (0.1 ml) of graded doses of sodium nitroprusside (0.4, 0.8, 1.6, 3.2, 6.4, 12.8, and 25.6 μg/kg; Sigma-Aldrich, United States) and phenylephrine (0.2, 0.4, 0.8, 1.6, 3.2, 6.4, and 12.8 μg/kg; Sigma-Aldrich, United States). These drugs were used to induce a decrease or increase in BP, respectively. Sodium nitroprusside and phenylephrine injections were given in a random order and subsequent injections were not given until the recorded parameters had returned to pre-injection levels. The cardiac BR function was analyzed using a logistic regression over the entire pressure range (Negrão et al., 1993; Michelini et al., 2003). Data was fit to the equation: HR = A/[1 + exp{B(MAP-C)}] + D, where A is HR range; B is the slope coefficient; C is the pressure at the midpoint of the range (midpoint BP); and D is the minimum HR. The peak slope (maximum gain) was determined by the first derivative of the baroreflex curve and was calculated with the equation: Gain = A(1) × A(2) × [1/4], where A(1) is the range and A(2) is the average slope. The mean values for each curve parameter were used to derive composite curves for each group of rats.

### Dose-Responses Analysis to BR Stimulation

To determine whether the effects of High-Altitude in BR control could be associated to differences in BP stimulus, we constructed a dose-response curve for SNP and Phe. We used 8 doses of SNP (concentration: 0.0512 μg/μL; at 0.1; 0.2; 0.4; 0.8; 1.6; 3.2; 6.4; and 12.8 μL/kg) and 8 doses of Phe (concentration: 0.1024 μg/μL; at 0.2; 0.4; 0.8; 1.6; 3.2; 6.4; 12.8; 25.6 μL/kg). The curve was constructed using the logarithm of different doses. The responses were estimated using the delta of MABP (ΔMABP) from previous baseline measurements.

### Autonomic Control

Heart rate variability (HRV) was used as an indirect measurement of autonomic balance of the heart (Del Rio et al., 2016; Andrade et al., 2017). The first derivative of the BP (Dp/dt) signal was used to calculate the HR. Autoregressive algorithm, after Hann windowing with 50% overlap, was used to obtain power spectral density of HRV. Cut-off frequencies were defined as low frequency (LF_HRV_): 0.04–0.6 Hz and high frequency (HF_HRV_) 0.6–2.4 Hz (Andrade et al., 2017). Additionally, we used LF/HF_HRV_ ratio as an indicator of autonomic balance of the heart. LF_HRV_ and HF_HRV_ were expressed as normalized units (n.u.). Analysis was performed within a 10 min window. This analysis was performed in LabChart 7.3.8 HRV module software (ADInstruments, Bella Vista, NSW, Australia). In addition, to estimate the autonomic contribution on BR function, spectral non-stationary analysis was used (2-s resolution). The HF_HRV_ component (0.6–2.4 Hz) was used as an indicator of parasympathetic modulation. This analysis was performed with Kubios HRV Premium Software V 3.1 (Kubios, Finlandia).

### Statistical Analysis

Data were expressed as mean ± standard error of the mean. All data were subjected to Shapiro-Wilk normality test. The unpaired t-test at two tails was employed to compare the differences between groups. *p* < 0.05 was considered statistically significant. Statistical analyses were performed by GraphPad Prism 8.0 (GraphPad software Inc., San Diego, CA, United States).

## Results

### Effect of High-Altitude Exposure on Baseline Physiological Variables

Baseline physiological variables for both groups are shown in Table 1. At baseline, there were no significant differences between Sea-Level and High-Altitude exposure on body weight DBP, SBP, MABP, PP, and HR (Table 1).

**TABLE 1:** Effect of high-altitude exposure on baseline physiological parameters.

	**Sea Level (*n* = 7)**	**High Altitude (*n* = 5)**	***p*-value**
Body Weight (g)	386.57 ± 7.67	355.01 ± 11.47	0.45
SBP (mmHg)	140.68 ± 2.35	145.28 ± 5.56	0.61
DBP (mmHg)	104.17 ± 3.57	107.22 ± 4.79	0.41
MABP (mmHg)	116.34 ± 3.02	119.91 ± 5.01	0.53
PP (mmHg)	36.51 ± 2.38	38.06 ± 1.51	0.63
HR (bpm)	353.64 ± 11.18	371.94 ± 16.91	0.37

*Values are mean ± standard error of the mean (SEM). SBP, systolic blood pressure; DBP, diastolic blood pressure; MABP, mean arterial blood pressure; PP, pulse pressure; HR, heart rate. Unpaired *t*-test.*

### Effect of High-Altitude Exposure on Cardiac Autonomic Control at Rest

**FIGURE 1:**
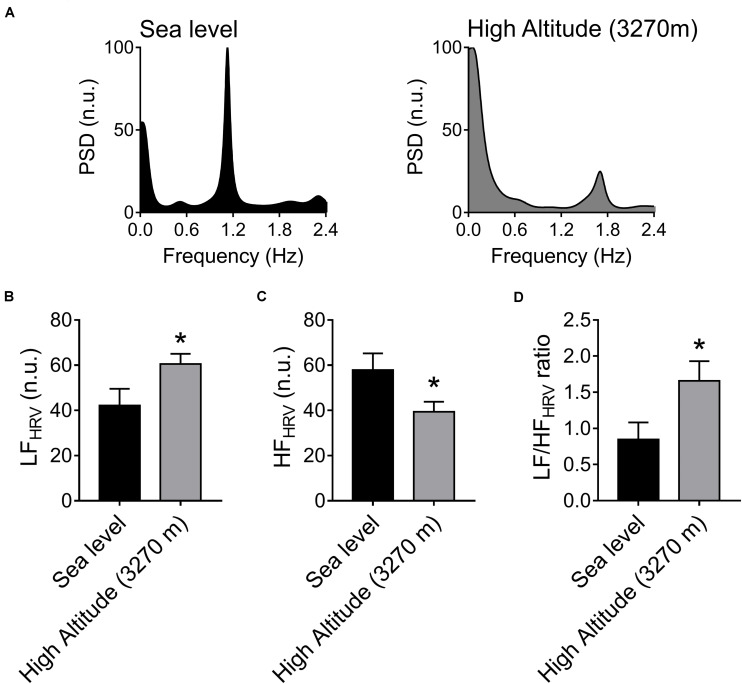
Effect of High-Altitude (3,270 m above sea level) on heart rate variability (HRV) alterations. **(A)** Representative heart rate variability (HRV) spectrums from one Sea-Level rat and one High-Altitude rat. During hypoxic natural environment HRV spectrum was altered in High-Altitude rats. **(B–D)** Summary of the effects of High-Altitude on **(B)** low frequency (LF) component of the HRV, **(C)** high frequency (HF) component of HRV and **(D)** LF/HF ratio. During High-Altitude exposure the animals displayed an increase of LF (**B**, sympathoexcitation) component, decrease of HF (**C**, parasympathetic withdrawal) component, and consequently an increase of LF/HF ratio of HRV **(D)**. Values are mean ± SEM. Data was analyzed by unpaired *T*-test. *p < 0.05 vs. Sea-Level. Sea-Level *n* = 7; High-Altitude, *n* = 5.

The autonomic control of the heart was estimated by HRV disturbances (Figure 1). After acute High-Altitude exposure rats displayed an increase of sympathetic drive and decrease of parasympathetic modulation of the heart (Figure 1). Indeed, the LF_HRV_ component was significantly increased (*p* < 0.05) from Sea-Level (42.12 ± 7.44 n.u.) to High-Altitude (60.55 ± 4.47 n.u.) (Figures 1A,B), while the HF_HRV_ component was significantly reduced (*p* < 0.05) from Sea-Level (39.37 ± 4.44 n.u.) to High-Altitude (57.82 ± 7.43 n.u.) (Figures 1A,C). Consequently, the LF/HF_HRV_ ratio was significantly increased (*p* < 0.05) at High-Altitude compared to Sea-Level (1.66 ± 0.27 vs. 0.85 ± 0.23, respectively, Figures 1A,D). In addition, our data did not reveal significant differences between Sea-Level and High-Altitude on RMSSD, SDNN, LF, and HF non-normalized units, total power, SD1, SD2, and SD2/SD1 ratio (Table 2).

**TABLE 2:** Effect of high-altitude exposure on heart rate variability parameters at rest.

	**Sea Level (*n* = 7)**	**High Altitude (*n* = 5)**	***p*-value**
RMSSD	2.17 ± 0.31	1.84 ± 0.19	0.43
SDNN	3.06 ± 0.31	2.27 ± 0.26	0.09
VLF (ms^2^)	1.36 ± 0.16	0.52 ± 0.11	0.01
LF (ms^2^)	3.39 ± 0.50	3.24 ± 0.74	0.20
HF (ms^2^)	3.93 ± 1.51	2.01 ± 0.47	0.28
Total power	6.24 ± 1.79	5.76 ± 1.20	0.08
SD1	1.53 ± 0.21	1.31 ± 0.14	0.43
SD2	4.02 ± 0.40	2.91 ± 0.35	0.07
SD2/SD1 ratio	2.79 ± 0.30	2.24 ± 0.19	0.19

*Values are mean ± standard error of the mean (SEM). RMSSD: Root-mean-square of the successive differences between adjacent normal R-R intervals; SDNN, Standard deviation of the normal-to-normal intervals; VLF, very low frequency component of HRV; LF, low frequency component of HRV; HF, high frequency component of HRV; SD1, short-term standard deviation from Poincare plots; SD2, long-term standard deviation from Poincare plots; SD2/SD1 ratio, of standard deviations of Poincare plots, Unpaired *t*-test.*

### Effect of High-Altitude Exposure on Cardiac Baroreflex Control

**FIGURE 2:**
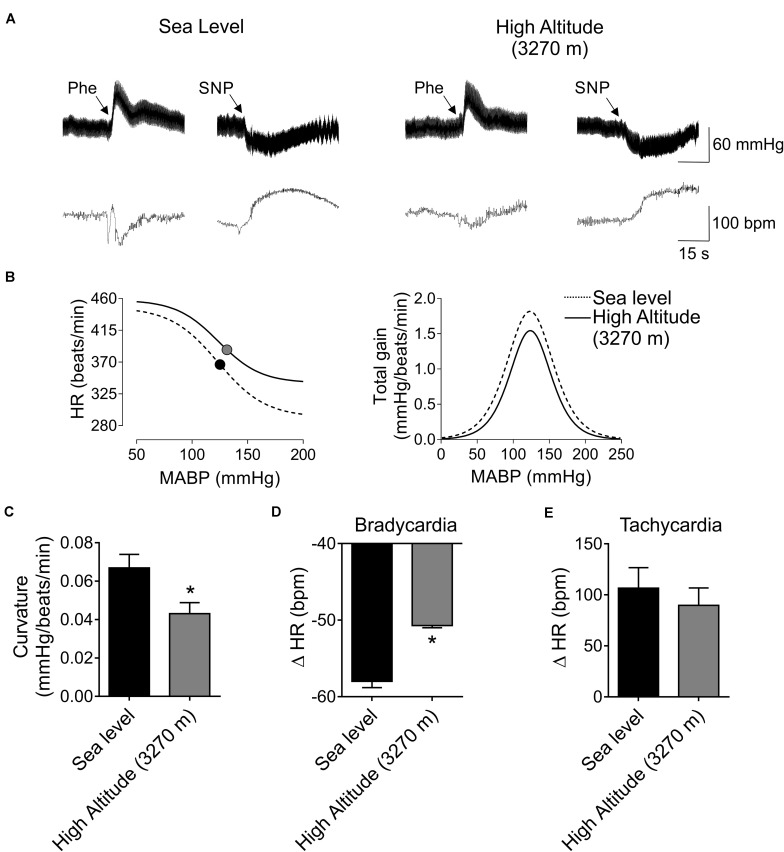
Effect of High-Altitude (3,270 m above sea level) on baroreflex (BR) control in freely moving rats. **(A)** Representative traces of blood pressure (BP) and heart rate (HR), during phenylephrine (Phe) and sodium nitroprusside (SNP) bolus, in one Sea-Level rat and one High-Altitude rat. Note that, during Phe there is a decrease of HR responses. **(B)** Summary curve of BR responses during Sea-Level and High-Altitude exposure. Note that maximum vagal bradycardia response was decreased during hypoxic natural environment. However, the maximum tachycardic responses was indistinguishable between Sea-Level and High-Altitude conditions. Total gain was not different between experimental groups. **(C–E)** summary data of curvature **(C)**, maximum bradycardia **(D)** and tachycardia responses **(E)**. Note that curvature and maximum bradycardic responses of BR function, were significant different between Sea-Level compared to High-Altitude exposure. Values are mean ± SEM. Data was analyzed by unpaired *T*-test. *p < 0.05 vs. Sea-Level. Sea-Level *n* = 7; High-Altitude, *n* = 5.

The BR parameters after High-Altitude exposure are shown in Figure 2 and Table 2. A representative recording of BP and HR after Phe and SNP administration at Sea-level and High-Altitude. After acute High-Altitude exposure and after Phe administration the bradycardic responses was significantly decreased (*p* < 0.05) (Figure 2A). Indeed, the sigmoidal curve of BR analysis, showed a blunted BR vagal bradycardia (Figure 2B). Moreover, the curvature (0.04 ± 0.01 vs. 0.07 ± 0.01 mmHg/beats/min, Figure 2C) and maximal bradycardia (50.70 ± 0.28 vs. 58.01 ± 0.81 beats/min, Figure 2D) were significantly reduced (*p* < 0.05) after acute High-Altitude exposure compared to Sea-Level. However, maximum tachycardic response to SNP, range, slope, midpoint of BP, lower plateau and upper plateau of BR analysis, were not significantly different between Sea-Level and High-Altitude groups (Figures 2A,E and Table 3).

**TABLE 3:** Effect of high-altitude exposure on baroreflex control.

	**Sea Level (*n* = 7)**	**High Altitude (*n* = 5)**	***p*-value**
Range (beats/min)	112.81 ± 11.93	122.80 ± 34.09	0.75
Slope (beats/mmHg)	1.88 ± 0.17	1.56 ± 0.11	0.20
Midpoint BP (mmHg)	135.81 ± 4.69	123.30 ± 5.43	0.12
Lower plateau (beats/min)	298.31 ± 13.74	338.90 ± 19.48	0.11
Upper plateau (beats/min)	411.10 ± 17.18	461.71 ± 21.16	0.09

*Values are mean ± standard error of the mean (SEM). Unpaired *t*-test.*

### Effect of High-Altitude Exposure on Parasympathetic Modulation of R-R Interval Time Series

**FIGURE 3:**
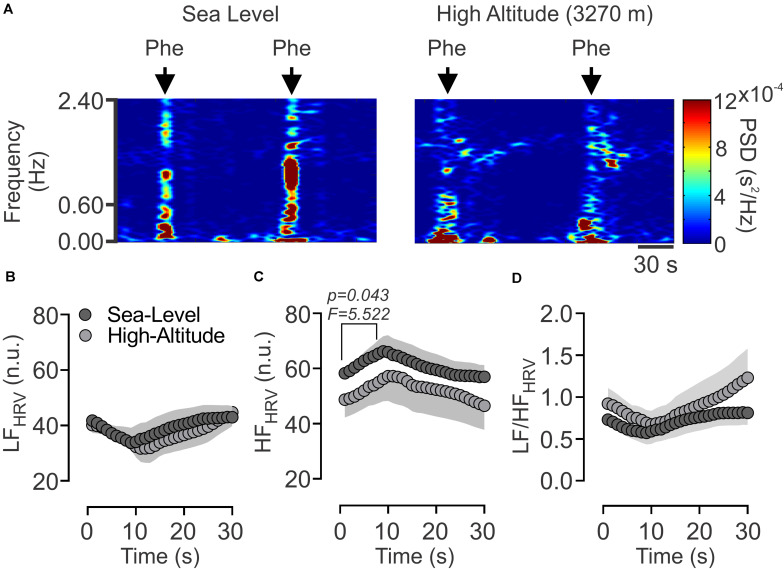
Effect of High-Altitude (3,270 m above sea level) on heart rate variability (HRV) alteration in time-varying domain, following phenylephrine (Phe) administration in freely moving rats. **(A)** Representative time-varying domain spectrum (2-s resolution) of heart rate variability (HRV) during phenylephrine administration (0.05 μg/mL) in one rat per group. Note that High-Altitude rat showed a reduced response to Phe between 0.60 and 2.4 Hz [high frequency (HF) component of HRV] related to parasympathetic control. **(B–D)** Summary of the effects of High-Altitude on **(B)** low frequency (LF) component of the HRV, **(C)** HF component of HRV and **(D)** LF/HF ratio during administration period of Phe. Note that between 2 at 8-s following Phe administration, the power spectral density of HF component of HRV **(C)**, was significantly reduced during High-Altitude exposure. Values are mean ± SEM. Two-ways ANOVA followed by Fisher *post hoc* test. Sea-Level *n* = 7; High-Altitude, *n* = 5.

Whereas there are very few evidences showing changes in parasympathetic and/or sympathetic outflow during natural High-Altitude environment. Considering current results showing a decrease of maximum vagal bradycardia induced by Phe, we determine if a diminished maximum parasympathetic activation occurs during acute High-Altitude exposure (Figure 3). The Phe administration (0.05 μg/mL) induces an increase of parasympathetic drive to the heart (0.6–2.4 Hz) (Sea level: 58.25 ± 2.30 vs. 65.89 ± 6.37 n.u., basal vs. Phe peak activation, respectively, *p* < 0.05) (Figures 3A,C), however, the vagal activation after High-Altitude exposure was reduced (High-Altitude: 48.69 ± 6.61 vs. 57.21 ± 9.15 n.u., basal vs. Phe peak activation, respectively, *p* < 0.05) (Figures 3A,C). LF and LF/HF ratio, were not different between groups during phenylephrine administration. In addition, in both conditions RMSSD, SDNN, SD1 and SD2 were significantly different (*p* < 0.05) between resting condition compared to SNP administration (Table 4). Regarding to Phe administration, Sea-Level group showed a significant decreased of non-normalized HF component of HRV (Table 4). VLF, LF, SD2/SD1 were no different between groups (Table 4).

**TABLE 4:** Effect of high-altitude exposure on heart rate variability parameters at sea level and high-altitude during phenylephrine (Phe) and sodium nitroprusside (SNP).

	**Sea Level (*n* = 7)**			**High-Altitude (*n* = 5)**		
	**Rest**	**SNP**	**Phe**	**Rest**	**SNP**	**Phe**
RMSSD	3.110.94	5.730.53*	2.000.31	1.910.32	5.400.88*	1.450.29
SDNN	3.600.97	8.861.91*	3.350.37	1.900.31	7.320.97*	2.490.43
VLF (ms^2^)	8.623.32	24.599.80	10.694.31	4.212.10	939.36705.90	5.341.98
LF (ms^2^)	21.365.86	65.3343.34	6.311.34	11.232.35	27.6010.47	5.773.26
HF (ms^2^)	16.284.29	14.383.41	4.141.30*	16.994.79	14.655.69	2.420.76
SD1	2.210.66	4.060.37*	1.420.22	1.350.22	3.830.62*	1.020.20
SD2	4.521.20	11.602.77*	4.450.52	2.280.42	9.391.42*	3.330.58
SD2/SD1	2.220.25	2.840.62	3.530.69	1.770.40	2.700.58	3.450.35

Values are mean ± standard error of the mean (SEM). RMSSD, Root-mean-square of the successive differences between adjacent normal R-R intervals; SDNN, Standard deviation of the normal-to-normal intervals; VLF, very low frequency component of HRV; LF, low frequency component of HRV; HF, high frequency component of HRV; SD1, short-term standard deviation from Poincare plots; SD2, long-term standard deviation from Poincare plots; SD2/SD1 ratio, of standard deviations of Poincare plots, *p-*value* < 0.05 vs. Rest. Two-way ANOVA.

### The Effects of High-Altitude Exposure on Baroreflex Control and Parasympathetic Modulation Are Not Dependent of Blood Pressure Stimulation

**FIGURE 4:**
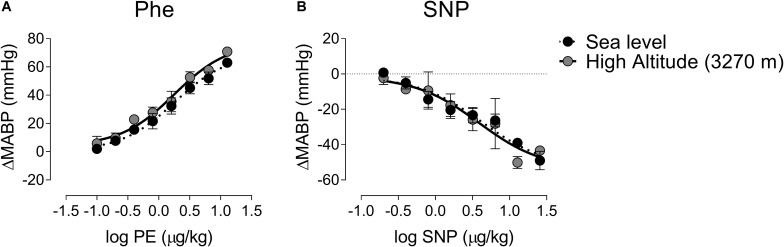
Dose-response curve of phenylephrine (Phe) and sodium nitroprusside (SNP) during Sea-Level and High-altitude (3,270 m above sea level) exposure. During Sea-Level and High-Altitude exposure were not found significant differences on mean arterial blood pressure (MABP) responses between Sea-Level and High-Altitude exposure, following Phe **(A)** and SNP **(B)** administration. Values are mean ± SEM. Two-ways ANOVA followed by Fisher *post hoc* test. Sea-Level *n* = 7; High-Altitude, *n* = 5.

To determine if differences observed during High-Altitude exposure are dependent of BP changes, we determine the dose-responses of BP at different concentration of Phe and SNP (Figure 4). Phenylephrine bolus administration produces a sigmoidal response in BP (Figure 4A). There are no significant differences in Log EC50 between groups (0.25 ± 0.07 vs. 0.25 ± 0.12 μg/kg, Sea-Level vs. High-Altitude group, Figure 4A) and *R*^2^ (0.99 ± 0.98 Sea-Level vs. High-Altitude group, Figure 4A). Similarly, the effect of sodium nitroprusside administration on BP was not significantly different between environments (Log EC50 = 0.63 ± 0.24 vs. 0.58 ± 0.24 μg/kg, Sea-Level vs. High-Altitude group; and *R*^2^ = 0.93 ± 0.94 Sea-Level vs. High-Altitude group, Figure 4B).

## Discussion

The major findings of the present study were: (i) after acute High-Altitude exposure there is a cardiac autonomic control impairment; (ii) exposure to High-Altitude produces a deterioration of cardiac BR control (vagal bradycardic responses and curvature) in normal rats; and (iii) there is a reduced activation of parasympathetic drive during acute High-Altitude exposure in normal rats. The present results suggest that acute exposure to high-altitude produces an autonomic control impairment and a deterioration of BR function, mainly characterized by parasympathetic withdrawal after 24 h of high-altitude exposure.

### Baroreflex Control and High-Altitude Environment

Estimations of the World Health Organization (World Health Organization [WHO], 1996) indicate that ∼140 million people in the World are living at high-altitude (≥2,500 m). It has been showed that during hypoxia there is a BR control impairment, however, some differences may be associated to atmospheric pressure. In fact, during hypobaric-hypoxia there is a reduce of BR gain, without modification of BR set point (Sagawa et al., 1997; Yazdani et al., 2016); but contrarily, normobaric-hypoxia did not modify BR gain (Sagawa et al., 1997). In addition, Obrezchikova et al. (2000), showed that exposure to hypobaric hypoxia (hypoxic chamber) inhibits BR vagal bradycardia in rats. Contrarily to these evidences, our data showed that during High-Altitude exposure (acute exposure), there are no modifications of BR gain either BR set point, which could be related to different levels of High-Altitude simulation (Sagawa et al., 1997; Obrezchikova et al., 2000; Yazdani et al., 2016), some interaction between environmental factors as well as related to some methodological differences (natural environments vs. simulated high-altitude; catheterization vs. indirect BP measurements; drugs administration vs. neck collar, among others). Also, our results strongly suggest that BR control could be affected by atmospheric pressure, therefore, showing that BR control impairment is not exclusively related to oxygen reduction. However, it has been very well demonstrated that hypoxia produces an increase of chemoreflex drive, which could be associated to a reduction of BR control (Del Rio et al., 2014). Therefore, it is possible that our results may be related to a chemoreflex potentiation and consequently a reduction of BR sensitivity (Del Rio et al., 2014). Along with this, previously we found that in a pre-clinical model of hypertension induced by chronic intermittent hypoxia, the BR control impairment was restored after peripheral chemoreceptor ablation (Del Rio et al., 2016). This suggest that at the central level, chemoreceptor neurons could modulate BR function. Indeed, it has been proposed that at the level of the nucleus of tracts solitaries (NTS), chemo and baroreceptor neurons interact with each other (Miura and Reis, 1972; Somers et al., 1991), which may partially explain BR impairment at high-altitude exposure. Nevertheless, our experiments cannot reveal if a chemoreflex-baroreflex interaction during High-Altitude exposure exists. However, despite that our data showed a robust effect of High-Altitude on parasympathetic modulation of BR function, we did not discard the possible role of atmospheric pressure on baroreflex control. Then, it is necessary to determine the possible contribution of the reduction of atmospheric pressure and the role of hypoxia, but in a natural environment.

### High-Altitude Exposure and Autonomic Control Impairment

It has been proposed that acute hypoxia is a potent activator of sympathetic drive in a time dependent manner (Rostrup, 1998; Hainsworth et al., 2007). Moreover, after acute hypoxic exposure there is an autonomic control impairment, increasing muscle sympathetic nerve activity and HRV disturbances (Cornolo et al., 2004). Indeed, using HRV analysis, healthy patients display an increase of LF/HF ratio and only the HF (n.u.) component of HRV was severely decreased after 24 h of hypoxic environment (Cornolo et al., 2004). Accordingly, Obrezchikova et al. (2000) also showed that during hypoxic challenges (hypobaric hypoxic chamber), the parasympathetic modulation is decreased in conscious freely moving rats. Similarly, it has been shown that chronic exposure to High-Altitude, causes increases in HR due to parasympathetic withdrawal (Dhar et al., 2014; Siebenmann et al., 2017). Something interesting to note, is that the parasympathetic withdrawal could persist after one month of high-altitude exposure (Farinelli et al., 1994; Dhar et al., 2014). Our results are in line with those obtained by the aforementioned studies. Indeed, our animals displayed an increase of LF, decrease HF and, consequently, increase of LF/HF ratio after 24 h of high-altitude exposure. Interestingly, it has been shown that plasma norepinephrine was decreased 2 days after high-altitude exposure and the increase was only observed at the third day (Rostrup, 1998). Thus, considering that after 2 days of high-altitude exposure the norepinephrine is decreased (Rostrup, 1998), it is possible that the HRV disturbances in our data may be related to parasympathetic modulation rather than sympathetic activation. Indeed, using non-stationary analysis, after 24 h of high-altitude exposure, it showed that the parasympathetic activation (phenylephrine) is decreased compared to the sea level condition. Therefore, the autonomic disturbances may be mainly related to parasympathetic withdrawal than to sympathoexcitation during high-altitude exposure.

In addition, despite that in our experiments we did not evaluate norepinephrine release, our results showed that BR maximum tachycardic response was not different between sea level and high-altitude exposure, suggesting that the sympathetic drive during high-altitude could not be related to BR control, if not to an interaction between peripheral chemoreflex activation and baroreflex control impairment, triggering parasympathetic withdrawal (Del Rio et al., 2016). Nevertheless, we did not evaluate chemoreflex response in our experiments, which might be related to autonomic control impairment observed in our data (Hayashida et al., 1996; Schultz et al., 2007). Thus, further research is needed to elucidate the possible relationship between baroreflex and chemoreflex and their role on cardiac autonomic function, but in a natural environment, which is characterized by differences in atmospheric pressure, humidity and in several territories with different temperatures.

In addition, from a practical perspective, our work could be applied to people exposed to “live low – work high” conditions. In several countries, (i.e., Peru, Chile, among others) the growth domestic product is critically dependent of bigger mining whose operations are mostly at high-altitude. Additionally, Chile is a country with an important number of astronomers (e.g., ALMA project) exposed to the aforementioned “live low – work high” conditions. Further, in several sports, competitions are celebrated at high-altitude (e.g., Olympic Games, Mexico 1968). Therefore, our results could transfer to people which are subjected to high-altitude exposure with a focus on improving baroreflex control and parasympathetic modulation of the heart, which could improve the functional capacity at high-altitude.

### Limitations

Regarding some potential limitations of our study, we did not use the same animals at Sea-Level and at High-Altitude conditions, which could mask some physiological responses to High-Altitude exposure. However, our results showed that after acute High-Altitude exposure our animals displayed several characteristics of hypobaric hypoxic exposure. In addition, our experiments were limited to 24 h post-High-Altitude condition. Thus, it is not possible to infer the long-term effects of High-Altitude exposure on cardiac BR control from our study. Moreover, we did not determine the contribution of ventilatory chemoreflex control, which could be related to BR control impairment after 24 h of High-Altitude exposure (Halliwill et al., 2003). In addition, we performed our physiological experiments 8 h after surgical catheterization, which could affect BR control (Tohyama et al., 2018), however, all animals were subjected to the same procedure, therefore, they should be comparable. Other potential limitation of our study, was the different relative air humidity during the two experimental conditions. Nevertheless, it is important to mention that the experiments were carried out in typical environmental condition to which it is subjected when a person is exposed to High-Altitude (Bruce-Low et al., 2006; Abellán-Aynés et al., 2019). Finally, we did not use pharmacological approaches to characterize sympathetic and parasympathetic contributions to cardiac BR control after high-altitude exposure.

## Conclusion

Cardiac autonomic dysfunction and BR control impairment occur after 24 h (i.e., acute) of High-Altitude exposure. In addition, the parasympathetic activation was decreased after high-altitude exposure. Therefore, the short-term exposure to high-altitude produces an autonomic control impairment and BR dysfunction, which could be more related to parasympathetic modulation of the heart rather than sympathetic drive.

## Data Availability Statement

The datasets generated for this study are available on request to the corresponding author.

## Ethics Statement

The animal study was reviewed and approved by University of Antofagasta Scientific Research Ethical Committee (CEIC-210/2019).

## Author Contributions

AB, AA-Á, MV-M, RR-C, MI, MR, RD and DA designed the work and contributed to analysis, interpreted the the data, and drafted the work. AB and DA performed the data collection and analysis and contributed to the concept of the project and experimental design. All authors approved the final version of the manuscript.

## Conflict of Interest

The authors declare that the research was conducted in the absence of any commercial or financial relationships that could be construed as a potential conflict of interest.
